# Teaching perspectives on the communication of difficult news of genetic conditions to medical students

**DOI:** 10.1002/ajmg.a.63003

**Published:** 2022-10-26

**Authors:** Ashley M. Vanasse, Tracey Weiler, Elizabeth A. Roth, Sharmila Upadhya, Helga V. Toriello, Ariel J. VanLeuven, John R. Norris, John C. Carey, Andrew K. Sobering

**Affiliations:** ^1^ Department of Biochemistry St. George's University St. George's Grenada; ^2^ Department of Pediatrics University of Oklahoma School of Community Medicine Tulsa Oklahoma USA; ^3^ Department of Human and Molecular Genetics Herbert Wertheim College of Medicine, Florida International University Miami Florida USA; ^4^ AU/UGA Medical Partnership Campus of the Medical College of Georgia Athens Georgia USA; ^5^ Department of Pediatrics and Human Development Michigan State University – College of Human Medicine East Lansing Michigan USA; ^6^ Department of Cellular Biology and Anatomy Medical College of Georgia, Augusta University Augusta Georgia USA; ^7^ Department of Pediatrics University of Utah School of Medicine Salt Lake City Utah USA; ^8^ Windward Islands Research and Education Foundation True Blue St. George's Grenada

**Keywords:** assessment, communication, difficult news, genetics education, unexpected news

## Abstract

Informing parents that their child has a diagnosis of Down syndrome (DS) is a common example of the delivery of unexpected or difficult news. Expectations and life planning will change, and if detected prenatally, discussions might include the option of pregnancy termination. Medical school curricula currently include training in breaking unexpected news; however, it is difficult to teach and assess. We use the perspectives of clinicians, educators, and a medical student who is the parent of a child with DS to frame a discussion on teaching, practicing, and assessing communication of difficult news in human genetics during medical school.

## 1 INTRODUCTION

An ongoing challenge in medicine remains the inevitable need to break so‐called “bad” news to a patient or family. The traditional term, “bad news” carries connotations that parents might not appreciate, especially in the context of Down syndrome (DS). Recent discussions suggest using the terms unexpected, or difficult news (Brasington, 2007). While there may be no perfect way to deliver the news, history and personal experience tell us there are many that are inappropriate (Dent & Carey, 2006). Additionally, evidence‐based reviews have been published to help guide both prenatal and postnatal discussions (Skotko, Capone, & Kishnani, 2009; Skotko, Kishnani, & Capone, 2009). The training, skills, and attitudes associated with discussing a DS diagnosis have been studied for residency students (Ferguson et al., 2006; Lunney et al., 2012). However, to our knowledge there are no reports in the medical literature discussing how this topic should be integrated and assessed in the context of genetics teaching during preclinical medical education. Here, we discuss strategies to develop this communication skill from various perspectives.

### 1.1 Parental and medical student perspective

Four years before starting medical school, I (AMV) became pregnant with twins and received a prenatal diagnosis that one of my boys would have DS. This diagnosis was initially relayed to me by my obstetrics and gynecology (OB‐GYN) physician. I was told that my life would be hard and to not expect much from my son. He would always live with me and would never do the typical things that other people do. He would never be employed, learn to drive, or have meaningful relationships. I was left with the idea that his life would be bleak and almost pointless.

My first reaction was heartbreak and fear of not knowing what the future held for him or my family. Was I capable of caring for a child with disabilities? What impact would this have on his unaffected twin, my marriage, and our extended family? I had these, and other questions surrounding the diagnosis. There was no good way to answer them—I was an emotional wreck.

After the initial shock wore off, I found support from other parents and a local DS association. I learned that the future suggested by the physician and the life I imagined for my son before the diagnosis were two possibilities along a spectrum. I met young adults with DS who had graduated from high school, attended college, could drive, live on their own, hold jobs, and have meaningful relationships. They were valued members of their families and communities.

In 2018, I began medical school, a venture fueled by issues discovered from having a son with DS. In my first semester of medical school, the human genetics course was scheduled. I was apprehensive when cytogenetics and aneuploidy would be discussed and was particularly concerned about how DS would be presented. I had a preconceived idea that the opinion held by my OB‐GYN would be revisited. Bracing myself to hear the same depressing story, I was pleased that the discussion of DS and the implications of its diagnosis in this course differed from my personal experience. The subject was approached with enthusiasm for helping people affected with DS or other genetic disorders. Without judgment or pity, it was implied that these individuals were part of our society. Unbiased facts were presented, and nondirective counseling was described.

### 1.2 Clinical perspective

We have a responsibility to our patients, parents, and families to discuss difficult topics with empathy and compassion. We should be sensitive to the learned and shared beliefs of the diverse patients we see, resulting in approaches tailored to each individual case (Reynolds et al., 2005). We must provide the facts needed for patients and families to make informed decisions. Failure to do so undermines the public trust in all physicians. However, in addition to providing facts, we must consider our word choices which affect the care we deliver (Ring et al., 2016). Parents who are expecting or already have a child with congenital anomalies will likely be vulnerable or stressed. How we discuss a medical condition with a family can have long‐lasting effects on their self‐perception, how they raise their children, and their interactions with the community at large. The profound emotional effects created by the words and actions used by the person delivering the unexpected news are often encoded as “flashbulb memories” because the triggered emotive connotations can be ingrained in individuals and families over a lifetime (May et al., 2020).

Unfortunately, many physicians learn how to deliver difficult news observationally (Dubé et al., 2003). This activity is suggestive of an unstructured aspect of the social cognitive theory of educational psychology where personal and environmental determinants affect behavior (Bandura, 2018). Ideally, similar future encounters will be embedded with self‐reflection of performance in previous situations. If the observed experiences are nondirective, respectful, and empathic, they will help to positively shape the next encounter. However, if the observed experiences are directive or disrespectful, and the self‐reflection is left to chance, the possibility of a poor outcome is increased. We all have likely had experiences where we thought that an encounter could have gone better, and we left with the hope that next time we will improve (Dent & Carey, 2006). The initial development of skills for communicating unexpected information should not be left to chance encounters with real patients and families experiencing unexpected situations. Rather, we must provide a scaffolded learning approach. Ideally, students should first observe the delivery of difficult news, followed by practicing these skills in simulated situations. Learners can then partner with experienced practitioners in real situations as they transition into practice. This situated learning follows the legitimate peripheral participation learning theory where medical students are newcomers in the community of clinical practice (Jackson et al., 2019).

Scaffolding training within a variety of patient‐physician encounters can improve effective communication. This approach has been beneficial to various types of clinical practice including family medicine and pediatrics (Berkey et al., 2018), oncology (Bumb et al., 2017), and human genetics (Witt & Jankowska, 2018). Tools exist to facilitate the process for residents and fellows (Wolfe et al., 2016). However, training in communication by asking the right questions, eliciting appropriate responses, practicing empathy, and delivering unexpected news should begin early in medical school. Importantly, students must also learn how to cope with their own feelings after these encounters (Paul, 2018). Successful scaffolding and effective modeling of the learner experiences will likely reduce the chance of suboptimal handling of future similar situations as students become autonomous physicians.

### 1.3 Educator perspective

Communication skills are taught in a variety of ways during preclinical medical education (Sanson‐Fisher et al., 2018). Developing best practices for teaching these skills is difficult (Deveugele, 2015), but it is important to begin early in medical school (Graf et al., 2020). Medical genetics naturally lends itself to teaching communication skills, and it is taught in a variety of ways across the curriculum including traditional lecture (Plunkett‐Rondeau et al., 2015), small group discussions, team‐based learning (Reimschisel et al., 2017; Thatcher et al., 2017), and “clicker” sessions using audience response devices (Hassumani et al., 2015).

From the beginning of medical school, we should strive to provide students with the tools to develop communication skills and empathy. Importantly, within our teaching material, we should endeavor to promote usage of professional language (Allanson et al., 2009) and concepts of nondirective counseling when describing genetic conditions and relaying information about their causes and prognoses (Latimer, 2007; Nance, 2017). We must also ensure that we provide complete information regarding a genetic diagnosis in a nonjudgmental and unbiased manner. It is incumbent on the healthcare professional to perform this task because members of a community association might not be familiar with the full range of possibilities and disease severity associated with a particular genetic condition. Therefore, clinicians have the responsibility to provide complete descriptions of what is known about the condition: from the mildest manifestations, to the most severe, and the likelihood of occurrence.

There are some relatively simple approaches to facilitate this process. For example, within a lecture or during a small group discussion session, students can be asked to imagine a scenario where a physician must communicate to parents that the illness in their child is explained by a molecular diagnosis. Students would then be asked to mentally script how they would deliver this information. In this way students have the initiative to identify their learning needs as they consider how they would relay difficult news to a family. As a newcomer to the community of practice, medical students might envision how they would like to hear this news if it were delivered to themselves. They can begin to itemize the ideas that are important to address, and then practice saying them aloud. In our experience, we have observed that students often find this task difficult. Variations on the subject are limited only by the imagination of the faculty and can dovetail with diverse aspects of the curriculum. Examples include:

1. Prenatal testing identifies a fetus with DS.
2. Newborn screening (NBS) identifies an infant who may have phenylketonuria (PKU).
3. An infant is diagnosed with Tay–Sachs disease.
4. Genetic testing identifies an adult individual with Huntington disease.
5. A patient receives a positive colon cancer screening test.

Likewise, practicing the delivery of difficult news can be done in a variety of different ways following the educational theory of deliberate practice (McGaghie et al., 2010). This includes role‐playing, active participation, and utilizing standardized patients. For example, two students can play the role of parents who have just been told that their newborn has been diagnosed with DS while a third student plays the role of the physician. The remaining students in the group observe and offer commentary after the exercise. Students can record themselves role‐playing the encounter for subsequent review. Simulation labs or standardized patients may also be used to practice these scenarios. Educational sessions with standardized patients have been used to successfully develop communication skills among students in genetic counseling, dentistry, and nursing programs by helping students to hone communication skills, reduce anxiety, and increase confidence when discussing sensitive topics (Kessler et al., 2021; Meyer et al., 2022; Ok et al., 2020). Utility is increased when students are involved in activities with experienced facilitators who can observe, provide support, and offer feedback. The ideal outcome is for novice learners to develop expertise through a combination of observation of effective delivery of difficult news, followed by cycles of deliberate practice with feedback and debriefing creating an experiential learning cycle (Yardley et al., 2012).

From our observations, effective communication regarding a positive NBS for PKU is relatively easy for novice students to craft. Since PKU may be effectively managed with dietary restriction therapy, students have a platform for discussing options. Students also learn how to discuss the difference between “screening” and “diagnostic” testing. In contrast, discussing Tay–Sachs or Huntington disease is typically more difficult for students to script, since there is no treatment available, and the outcome is inexorable decline. Facilitators can alter the scenario by simulating feelings in the participants, such as guilt in parents from a high‐risk group who did not pursue carrier screening.

Informing parents of a prenatal DS diagnosis has its own challenges, including how to best frame the ramifications to the parents, offering various options, and avoiding the temptation to make personal decisions for the parents. Providing students with opportunities to mentally script responses, and practice saying them aloud, helps to develop skills needed for navigation of future encounters with vulnerable patients and parents. Recordings can be played in larger group meetings where students may discuss how it felt to deliver or receive the diagnosis. Asking questions such as “what went well?”, or “what could be improved?” are an important part of the larger discussion.

As follow‐up, students may write about their experiences, allowing the activity to become a narrative medicine exercise and an aspect of the hidden curriculum that empowers students as they become aware of its presence (Lawrence et al., 2018; Neve & Collett, 2018; Zaharias, 2018). Writing exercises require students to internally reflect on their experiences as they develop into healthcare professionals using new communication tools that allow effective caregiving for future patients and families (Soni & Eidelman, 2021). We posit that beginning the practice of communication skills in the first year of medical school is important to set the stage for medical students to develop more effective and empathetic discussions with their future patients.

### 1.4 Assessments and examinations

In addition to teaching communication skills and providing opportunities to practice discussing unexpected, sensitive, or difficult news, we must also assess these activities because testing is an inherent component of curricular design and learning (Cook & West, 2013; Scott, 2020; ten Cate, 2001). The delivery of difficult news specifically, and communication skills in general, are critical competencies and should be assessed during formative, summative, and objective structured clinical examinations. Because testing drives learning, inclusion of these elements in a variety of assessments will ensure that faculty address these skills within the curriculum, and that all students will spend time gaining expertise in this area.

The US National Board of Medical Examiners retired question samples for Step 1 includes items focused on communication, albeit all are not necessarily oriented to delivery of difficult news, nor specifically focused on genetics. From a review of the February 2021 released sample problems, 7 out of 119 multiple choice question (MCQ) items are formatted to test communication skills (https://www.nbme.org/, accessed September 01, 2021). We encourage medical educators to incorporate similar items, assessing communication competencies in formative and summative assessments. An example MCQ item, focused on the delivery of difficult news around a prenatal diagnosis of DS is shown below.

Example item:

A 24‐year‐old woman at 12 weeks gestation comes to the physician for a follow‐up visit to discuss prenatal testing results. Noninvasive prenatal screening by sequencing of cell‐free DNA from maternal circulation shows that the fetus has a high probability to have an aneuploid condition. Cytogenetic analysis of chorionic villi confirms a DS diagnosis. After informing her of the diagnosis, which of the following statements is most appropriate for the physician to use when discussing the test results?

- “After you and your husband discuss the results, we can schedule a follow‐up appointment.”
- “I'm sorry to give you this news, I wish I could have told you the baby is normal.”
- “Let's take some time now to discuss what these results mean and the options available.”
- “We really should order a follow‐up test to confirm this result.”
- “You should consider terminating the pregnancy since raising a child with DS is difficult.”

#### 1.4.1 Rationale and item explanation

A thoughtful and well‐written rationale provides additional teaching opportunities for students. Of the answer choices provided in the example it would be best to immediately discuss the results and options that are available to the pregnant woman without conveying judgment about the condition (answer C). Students should also be aware that there might be an equally correct, or possibly better course of action that is not listed in the answer choices provided. Indeed, it might be best for the physician to pause, provide space, and allow the expectant couple to direct the next direction of the discussion. To get at this, the course of action would be to acknowledge that a lot of information was relayed, inquire how the parents are feeling, and ask if they have questions.

It is not optimal to delay the encounter since the clinician needs to address the diagnosis and convey the news to the expectant parents. The parents do not need sympathy in this context, and offers to apologize, or saying “I'm sorry” should be avoided. Instead, the news should be discussed in a nonjudgmental, empathic, and honest manner. It is well understood that the clinical validity of cytogenetic analysis is exceptionally high, and it is inappropriate to offer a repeat test that is highly likely to give the same result while at the same time suggest false hopes of a testing error (Burke, 2014; Katsanis & Katsanis, 2013). Furthermore, it is not appropriate to advise maintaining or terminating the pregnancy as both of these suggestions contradict the concept of nondirective counseling and attempt to force the values of the physician onto the patient. Additional sample learning objectives, lead in phrases, multiple choice questions, and discussion points are included (Data S1).

#### 1.4.2 Resources for students

Numerous resources exist for students as they train to handle encounters with parents regarding a DS diagnosis. For instance, practice guidelines for genetic counselors (Sheets et al., 2011) outline recommendations for communication, recurrence probabilities, and community resources. Online sites such as *Operation House Call* (https://thearcofmass.org/ohc/; accessed May 01, 2022) and *Lettercase* (https://www.lettercase.org/education/medical-professionals/; accessed May 01, 2022) inform this topic. *Lettercase* has scaffolded modules starting with didactic learning that include open‐ended questions to help students clarify their thoughts in this area. Healthcare professionals then model encounters in case studies. Learners are encouraged to consider how they would respond. They choose between options, are provided with feedback, and then can view short videos to observe how an experienced practitioner communicates with parents in the encounter. In addition to providing didactics, websites such as *Operation House Call* can help to organize immersive experiences for medical students via visitation with individuals who have DS or other genetic conditions. This situated approach can engender an experiential learning environment via authentic interactions (Yardley et al., 2012). Other learning materials based on interactive media sources have been described (Ferguson et al., 2006; Lunney et al., 2012).

## 2 DISCUSSION AND SUMMARY

Delivery of an unexpected diagnosis should be done in a sensitive manner. Prior to conception it is unlikely that any couple expects to have a child with a congenital anomaly, and receiving this news is likely to be a surprise. In addition to the content, the wording, nonverbal and situational approach used by the physician when conveying a difficult diagnosis might be ingrained in the memory of the family forever. It is essential to use person first language (Crocker & Smith, 2019). For example, it is proper to say: “this child has DS,” whereas it is inappropriate to say, “This is a DS baby,” or “this child is Down's” (Noble et al., 2017). Physicians should avoid ascribing a negative sentiment toward the diagnosis and remain neutral. Beginning conversations with, “I have some bad news,” implies negativity. Alternatively, the phrase, “I have results to share with you,” allows the conversation to begin with a neutral tone. When discussing a diagnosis, students must learn how to give accurate and current information to provide patients with the knowledge needed to manage their new situation. For prenatal diagnoses, information on all possible options, including issues related to the pregnancy, the reproductive options, and the scope of the child's condition should be given. The importance of this approach is reflected by recent attempts to codify some aspects of this discussion with legislation to encourage conveyance of accurate facts (Lehman et al., 2021).

Providing sentiment that displays sorrow or sympathy should be avoided as this reinforces negative connotations (Matthews et al., 2019). In contrast, maintaining empathy and providing support to the family are beneficial (Sinclair et al., 2017). Appropriate discussions are important because parents who have children with congenital conditions are more likely to experience increased stress compared to parents who have typically developing children (Smith et al., 2014). Guidelines emphasize the importance of providing patient families with resources regarding diagnosis and support group opportunities (Skotko, Capone, & Kishnani, 2009). Surveys show that mothers who receive a prenatal DS diagnosis want up‐to‐date information provided at their clinical encounter. Additionally, they want information to help connect with appropriate support groups (Skotko, 2005).

We recommend early training in medical school to provide students with opportunities to observe and develop skills and strategies for discussing difficult topics. The objective is to allow students to progress from observing at the periphery, to practicing in a low‐stakes environment, and finally to effectively communicate in real‐world situations. Patients, parents, families, physicians, and the healthcare system in general all benefit when a diagnosis is delivered in an empathetic manner that is informative, nonjudgmental, and nondirective.

## AUTHOR CONTRIBUTIONS

All authors contributed to the development of this piece. The first draft of the manuscript was written by Ashley M. Vanasse and Andrew K. Sobering. All authors contributed to and commented on the many versions of this manuscript. All authors read and approved the final piece.

## CONFLICT OF INTEREST

Helga V. Toriello is an item writer for the NBME and NBOME and has recused herself from writing and commenting on the sample items included in this piece. The other authors have no competing interests to declare.

## Supporting information


**Appendix S1** Supporting Information

## Data Availability

Data sharing is not applicable to this article as no new data were created or analyzed in this study.
