# Supplementary material for: Teaching perspectives on the communication of difficult news of genetic conditions to medical students
Source: Am J Med Genet A. 2022 Oct 26;191(1):299–305. doi: 10.1002/ajmg.a.63003 (PMC10092636; doi:10.1002/ajmg.a.63003)
Supplement: Supplementary file 1 — Appendix S1 Supporting Information [file AJMG-191-299-s001.docx]

**Teaching in medical genetics to provide perspectives on communication of difficult news**

**Contents**

1. Caveat
2. Sample learning objectives for communication of concepts related to genetics content
3. Proposed examples of question “lead in” phrases for items.
4. Examples of MCQ items with discussion points.
5. **Caveat**

The authors recognize that the learning objectives, lead-ins, test items, and discussion points included in this supplement are not comprehensive, nor perfect, and flaws exist. We include this supplement to serve as a starting point to provide ideas for educators in medical genetics to spark the creation of other learning objectives and teaching tools in this area, along with development of other assessment items. Our goal is to encourage inclusion of these types of learning objectives into the medical student curriculum and suggest ways to assess them. The test items we provide might also be useful in student teaching sessions to foster discussion. It is the intention of the authors to collaboratively develop a more comprehensive set of learning objectives, lead-ins and test items amongst ourselves and in future educational workshops and conferences.

1. **Example Learning Objectives**

- Communicate the meaning of a variant of unknown significance to a patient.
- Describe abnormal findings to a patient in a calm, fact-based manner.
- Develop a patient-centered treatment/management plan for a patient affected with a genetic disorder to optimize patient compliance/adherence.
- During a patient education session focused on a genetic condition, provide balanced information about possible options and outcomes.
- Elicit information from a patient regarding family history for the purpose of creating a 3-generation pedigree.
- Elicit information from a patient regarding relevant family medical history in a sensitive, respectful and empathetic manner.
- In a genetic counseling session, provide information in a non-directive manner that is sensitive to the values and beliefs held by the patient.
- Provide context to genetic testing results, when associated with reduced penetrance, variable expressivity, anticipation, locus heterogeneity, or other genetic principles.
- Provide information to a patient regarding their specific pharmacogenetic variants and how that impacts their medication dose and schedule.
- Recognize desirable and undesirable qualities in communication of difficult news including non-directive versus directive counseling and empathy or compassion versus sympathy.
- Use non-judgmental language to inform a pregnant woman that her fetus is likely to have a congenital condition, disorder, or malformation.
- When a patient shares their direct-to-consumer genetic testing results, help the patient understand what these results mean, and what the next steps are in confirming the results.

1. **Example lead-in ideas might include the following:**

- Which of the following is the most appropriate initial response by the physician?
- Which of the following is the most appropriate opening remark by the physician?
- Which of the following is the most appropriate response by the physician?
- Which of the following is the most appropriate statement by the physician at this time?
- Which of the following is the most important point to raise during this encounter?
- Which of the following is the next most appropriate physician statement?
- Which of the following options best describes the flaw/error in the physician’s statement?
- Which of the following options best describes the physician’s response?
- Which of the following statements best conveys the recurrence likelihood in this situation?
- Which of the following statements is most likely to convey empathy?
- Which of the following statements would best communicate the implications of these findings to the parents (or to the patient)?
- Which of the following strategies is most appropriate to use in interpreting these testing results?
- Which of the following strategies is most likely to elicit an informative family history?

1. **Sample questions**
2. A 17-year-old girl is brought to the physician by her father for a routine physical examination, as she prepares to move away for college. She has normal intelligence, and is excelling in high school. She has disproportionate short stature with particularly short upper limbs and a prominent forehead. Both of her parents are of normal stature. Previous genetic testing shows that her condition is caused by a known pathogenic variant leading to increased signaling through the *FGFR3* tyrosine kinase receptor. Which of the following is the most important point for the physician to raise during this encounter?
3. Although the patient’s stature is reduced, mental acuity remains unaffected
4. It is important for everybody in the family to be happy and healthy
5. The father should pursue paternity testing since he has normal stature
6. The implications of achondroplasia for pregnancy and recurrence risk of the disorder
7. The patient has reached her maximum height; she will not grow any taller as she ages

Answer D

Rationale:

- The patient has achondroplasia, and she is of childbearing age. Before she becomes pregnant, it is important that she understand that she has a genetic condition that can be inherited in an autosomal dominant manner, and her children will have a 50% chance of also being affected. There are also special considerations for pregnant women with achondroplasia such as a possible requirement for a cesarean section for delivery because of a small pelvis size.
- With achondroplasia, it is not appropriate to compare intelligence with physical manifestations as the two are not connected.
- While a happy and healthy family is a good thing, this visit needs to focus on the patient.
- The patient probably already understands that she will not attain further height as she ages; this is not the most pressing concern at the moment.
- It is not surprising that an individual with an autosomal dominant disorder has parents who are not affected. A significant number of individuals with achondroplasia have parents who are of normal stature and the *de novo* causative genetic variant is formed during spermatogenesis. In any case, it is not appropriate for a physician to suggest paternity testing to a parent during a consultation with an affected child, but the physician should be fluent in genetics before answering should a parent ask for advice.

1. A 10-year-old boy is brought to the physician by his parents for a regular follow-up examination because of the progressive development of café-au lait spots and 12 neurofibromas distributed on his body. Genetic testing shows a germline pathogenic variant in *NF1*. This variant is not detected in DNA obtained from leukocytes of either parent. Which of the following is the most important statement for the physician to say to the parents during this patient encounter?
2. “Cosmetics might help to hide the dark café-au-lait patches on his skin.”
3. “His neurofibromas will likely become more numerous, larger and cause disfigurement.”
4. “If your son has children, their risk of being affected is 50%”
5. “Luckily there are no neuropsychiatric aspects to your son’s disorder”
6. “We will schedule annual follow-up visits because of increased cancer and psychiatric risks.”

Answer E

Rationale:

- Benign tumors that develop from cells and tissues that cover nerves are called neurofibromas which are the hallmark of neurofibromatosis (NF1). Individuals who have NF1 are also at an increased risk to develop various types of cancer during their lifetimes and are recommended to have regular follow-up consultations to monitor this risk. Individuals with NF1 are also at an increased risk to develop psychiatric complications – and this should be discussed with the parents.
- It is inappropriate to indicate that there are no neuropsychiatric aspects to NF1, as these are a part of the NF1 spectrum of disease.
- It is be inappropriate to bring up the topic of cosmetics for a 4-year-old, but if a parent makes the inquiry, then there is a compelling enough reason to discuss it.
- Some patients with NF1 will have progressive accumulation of neurofibromas and other manifestations, others might not. With NF1, this relatively extreme variable expressivity can be found, even in the same family. There is no way to predict who might have severe or progressive features.
- While it is true that the future children of this boy will have a 50% risk of being similarly affected, he is still many years away from puberty, and the more pressing concerns are monitoring for the development of cancer and psychiatric disorders.

1. A newborn girl has cleft lip and palate, microcephaly, malformed kidneys, and a congenital heart defect. Testing by rapid FISH shows that she has trisomy 13. When discussing the diagnosis with the parents and its implications for the care of the patient, which of the following is the most appropriate statement from the physician?
2. “Here is the test result, let’s schedule an appointment later this week to discuss further.”
3. “Like trisomy 21, most cases of trisomy 13 are caused by maternal meiotic nondisjunction.”
4. “The differences we see in your daughter are caused by an extra copy of chromosome 13.”
5. “We should avoid performing heart surgery since there are many other problems.”
6. “We should obtain a karyotype to find out if the baby has a Robertsonian translocation.”

Answer C

Rationale:

- The parents know that their child has congenital malformations, so at this point (of the options offered), they must be told that the rapid FISH test was positive for trisomy 13, and what that means.
- It is not appropriate to schedule an appointment for a later discussion as the medical issues posed by their child need to be addressed immediately, not later in the week.
- While it is true that most forms of trisomy 13, 18, and 21 are caused by meiotic nondisjunction events, it is not important for the parents to understand this detail. It is also not important for them to understand that a maternal non-disjunction event was the most likely cause. Neither of these facts help them to understand the disorder in their child and emphasizing the likelihood of a nondisjunction event during oogenesis has the possibility of focusing guilt on the mother.
- The nature of the heart defect along with the other manifestations of the syndrome need to be discussed with the parents; after that discussion a management plan can be created.
- It is indeed a good idea to eventually pursue a karyotype to rule out a Robertsonian translocation. Ruling out a translocation as the cause of the Patau syndrome is important during the discussion of recurrence risk with the parents, but this is not relevant for the immediate care of the patient.

1. A 36-year-old woman at 12 weeks gestation comes to the physician for a follow-up examination to discuss results from prenatal testing. A karyotype from cells obtained via chorionic villus sampling shows that her fetus has trisomy 2. Which of the following is the most appropriate statement for the physician to use when discussing the implications of these testing results for the child?
2. “Raising a child with DS is similar to any other child, there will be ups and downs.”
3. “Some individuals with DS can be severely affected, while others have a milder disorder”
4. “You are lucky it’s trisomy 21 and not trisomy 13, at least your child is likely to survive.”
5. “You can find plenty of community support to raise a baby who has Down syndrome.”
6. “You should carry this child to term since pregnancy termination is wrong.”

Answer B

Rationale:

- In many genetic disorders a wide distribution of severity may be observed; DS is not an exception, and some individuals may be severely affected, and others may have a much milder form of the condition.
- Of course, parents who raise any child will have good and bad days, but it is generally accepted that children with DS are likely to have special needs beyond those of an unaffected child. Parents should know to expect this.
- It is inappropriate to compare survivability of different genetic disorders; here the parent needs to understand the condition that her fetus has, not some other disorder for comparisons.
- It is true that there are community support groups for raising children who have DS and these groups might be valuable resources for parents. However, it is incumbent on the clinician to explain the range of possible outcomes and give the up to date medical advice pertaining to the condition.
- Nondirective counseling should be employed, and the personal viewpoints of the physician should not be provided to the patient, or parent. It is not appropriate to suggest to the parent that they should consider pregnancy termination, or avoid it; instead, each of the medical options should be discussed.

1. A 3-year-old boy is brought to the physician by his parents because of a 2-year history of progressive neurological deterioration. After his first year, he lost previously acquired skills including turning over, unsupported sitting, and crawling. Laboratory testing shows he is homozygous for a 4-bp insertion in exon 11 of the *HEXA* gene causing lysosomal accumulation of GM2 gangliosides. The couple is of Ashkenazi Jewish ancestry, and when they first got together, they did not obtain genetic screening for the most common genetic disorders found in the Ashkenazi population. They are now planning on having another child. Which of the following statements conveys the most important concept for the physician to communicate to the parents during this encounter?
2. “If your next child is unaffected, there is a 2/3 chance that it will be a carrier.”
3. “In many cases, allele homozygosity implies that the parents are somehow related.”
4. “If you choose *in vitro* fertilization to get pregnant, preimplantation diagnosis can test for this disorder.”
5. “Drugs are being developed to block nonsense-mediated decay caused by the frameshift variant.”
6. “Tay-Sachs disease is a very severe genetic disorder and you really shouldn’t consider additional children.”
7. “Your first child would not have been affected if you had initial genetic testing.”

Answer C

Rationale:

- The parents should be aware that assisted reproduction technologies exist that may help reduce the risk that a subsequent child will be affected.
- It is true that if this couple has another child that is unaffected it will have a 2/3 risk of being a carrier, but that should not be the main message for this couple because a carrier of the *HEXA* pathogenic variant is unaffected.
- In some cases, consanguinity may be the cause of homozygosity. However, homozygosity may also be due to a high carrier frequency with low allelic heterogeneity in the population such as the 4 bp insertion allele which accounts for approximately 70% of all pathogenic *HEXA* variants in the Ashkenazi Jewish population.
- Researchers may indeed be investigating the mechanism of nonsense mediated decay, but it is a complex process, and there are no approved therapies at the moment, so it is inappropriate to discuss and raise false hope with parents of an affected child. Worse, even if it was available, avoiding nonsense mediated decay would likely not be useful here because of the codon changing that would occur after the frameshift. Additionally, the typical lay person is unlikely to understand the science jargon of nonsense-mediated decay, and types of mutations so this discussion is not appropriate.
- Directive recommendations regarding reproduction are not useful for this couple. Nondirective counseling should be employed
- It is not constructive to blame the parents (and place guilt on them) for having a child with a genetic disorder regardless of why they did not obtain genetic screening for the most common pathogenic alleles in the Ashkenazi Jewish population.

1. A 42-year-old woman comes to the physician after receiving a positive pregnancy result from an over-the-counter test kit. The pregnancy is confirmed in the clinic and gestational age is estimated to be 12 weeks. Testing fetal cells by chorionic villus sampling and amniocentesis is discussed. Which of the following best describes the clinical utility of these prenatal tests?
2. Deciding whether to arrange for adoption
3. Discussing pregnancy termination with the mother
4. Identifying risk factors for genetic disorders
5. Obtaining medical care for the mother and the fetus
6. Preparing the mother for potential bad news

Answer D

Rationale:

- Of the choices offered, the most important reason for prenatal testing is to ensure that appropriate medical care can be organized for both the mother and the fetus. It is already well established that pregnancies in woman who are older than 35 are at increased risk for chromosomal trisomy conditions, such as Down, Edward, or Patau syndromes with Down syndrome being the highest risk. Knowledge of the presence of one of these disorders in a developing fetus is useful for managing the pregnancy and the delivery should the woman choose to continue with the pregnancy.
- The other options all contain elements of directive counseling in which the physician is implying what the parents should do, and which decisions should be taken.
- Discussions regarding maintaining the pregnancy, termination, or adoption are secondary to ascertaining a genetic disorder in a fetus.
- Obtaining a diagnostic test is in and of itself not part of the preparation process for a family or an individual to receive potential unexpected or difficult news.

1. A newborn boy born to a 26-year-old woman is noted to have features that are suggestive of a genetic syndrome including moderately diffuse hypotonia, up-slanted palpebral fissures, single palmar creases on both hands, and increased gap between the great and second toes on both feet. He has a grade 3 heart murmur. Which of the following is the best next step in this situation?
2. Ask if the infant resembles anyone else in the family
3. Suggest that the parents meet with their pediatrician to obtain the diagnosis
4. Discuss the baby’s features and their possible implications with the parents
5. Obtain a karyotype before raising any concerns
6. Screen for organ dysgenesis with a whole-body CT scan

Answer C

Rationale:

- The features of this baby are suggestive of trisomy 21 and this needs to be discussed with the parents in a nondirective, and nonjudgmental way. Within this discussion, testing and confirmation by chromosomal analysis can be offered. A rapid FISH test can also be used to quickly confirm the clinical DS suspicion (often within a day) because cells do not need to be cultured to obtain the result; instead, interphase stage cells can be probed with DNA sequences that hybridize to chromosome 21 to determine aneuploidy. Typically, rapid FISH tests for the 3 known chromosomal aneuploid conditions (13, 18, and 21) and the sex chromosomes. A karyotype takes approximately three days to complete because blood leukocytes must first be cultured to isolate cells in mitosis. Even if there is a positive result from rapid FISH, a karyotype is usually sought at some later date to verify whether the underlying chromosomal aberration is structural (translocation), or numerical (trisomy). The karyotype is important because it can inform recurrence risk estimations.
- It is not appropriate to ask the parents about resemblance to other family members because the collective dysmorphological features are strongly suggestive of a genetic syndrome.
- A formal diagnosis should not be referred to another physician (primary care or otherwise) as it will incur an unnecessary delay.
- The parents may be told that there is a suspicion of a genetic disorder, and it will be tested for, but the implications do not need to be discussed unless there is confirmation.
- A computed tomography scan is a source of ionizing radiation and should not be sought unless there is a compelling reason to do so.

1. A 4-year-old girl is brought to the physician by her mother for a yearly checkup. Her development is slightly delayed and she has moderate attention deficit hyperactivity disorder. She has short palpebral fissures, thin upper lip, and a smooth philtrum. Which of the following represents the best next step in the evaluation of this patient?
2. Accuse the mother of alcohol abuse during the pregnancy
3. Ask the mother about past alcohol or substance use
4. Discuss the physical findings and their implications
5. Report the mother to child protective services
6. Suggest genetic testing for the child

Answer C

Rationale:

- The vignette describes a young child who possibly has fetal alcohol spectrum disorder (FASD). A frank but nonjudgmental discussion about the current physical findings and their possible implications opens the door to develop an appropriate care plan for the child and potential substance abuse treatment for the mother. By starting the conversation with the focus on the child, potentially productive discussions regarding prenatal exposures might still occur.
- It is unlikely that a positive outcome will result from accusing a mother of potentially harmful past behaviors.
- Likewise, beginning the discussion with questions about behaviors during pregnancy, or obtaining detailed social history is also not the next best step because in this case the patient is the child, not the mother.
- There is some evidence that genetic testing might be useful in FASD, however, the most pressing concern is management of the child. An initial focus on genetic testing simply delays relevant discussions.
- At this point it is not warranted to notify child protective services as the current issues result from past behaviors which may or may not be present and currently problematic.

1. A 45-year-old man comes to the physician after learning that his 71-year-old mother was diagnosed with Huntington disease (HD). He is concerned about these findings and wants to know what this means for his family and himself. Which of the following statements would be the most appropriate way for the physician to open the conversation about HD with this patient?
2. “HD is a progressive neurodegenerative condition and we can only provide symptomatic care.”
3. “HD is a pretty rare condition. Can you tell me what you know about it?”
4. “My condolences. I am so sorry to hear about your mother; this is a very difficult disease”
5. “I assume you want to know if you will also develop HD. I can arrange genetic testing”
6. “We should consider testing you and your children for family planning purposes”.

Answer B

Rationale:

- The best way to open this conversation is to follow the first part of the “ask-tell-ask” model to determine what the man knows about the condition. Afterward, more questions can be asked to determine what the patient is concerned about.
- Offering condolences is a way of expressing sympathy about the condition which provides or reinforces negative connotations about the disease. It would be better to express empathy for what the man might be feeling instead.
- It is better to not jump straight into some facts about the condition without determining what the patient knows or is interested in finding out.
- It is not appropriate to assume that the patient is interested in knowing more about his own risk. It is important to first ensure that the man understands the nature of HD, how it is managed, and his risk of having the disease himself.
- Additionally, it is inappropriate to begin discussing testing for the children of the patient for the same reasons.

1. A 24-year-old woman comes to the physician because she is concerned that her 65-year-old father was recently diagnosed with myotonic dystrophy. Laboratory testing shows her father has a pathogenic CTG expansion in the 3’ untranslated region of *DM1* and she is also interested in obtaining the test. She is concerned that if she has the mutation, she may have passed it on to her 2-year-old daughter. Her grandfather (now deceased) had clinical features of myotonic dystrophy late in his life, but he did not have the genetic test. Which of the following is the most appropriate statement for the physician to say during this discussion?
2. “Don’t worry because there is only a 50% chance that you may develop the disorder”
3. “Let’s not worry now because myotonic dystrophy typically affects people late in life”
4. “Many individuals have very mild features of the disorder and you may be one of them”
5. “There is a very minimal chance that your daughter has inherited this condition”
6. “Presymptomatic testing is available; let’s discuss the pros and cons for you and your family.”
7. “You can assume that you will be normal until you develop symptoms of the disorder”

Answer: E

Rationale:

- Presymptomatic testing is available but knowing the result typically comes with a heavy emotional toll. Advanced knowledge that a progressive neurodegenerative disorder will occur (such as Huntington disease or myotonic dystrophy) increases risk for depression and suicide. Learning that one will not become affected may improve an individual’s quality of life, but may also have adverse effects such as survivor’s guilt.
- Myotonic dystrophy manifests in the heterozygous state and is inherited in an autosomal dominant pattern which explains why there is a 50% recurrence risk in the children of an affected parent.
- Because the woman is not yet confirmed to carry the pathogenic variant, at this point, the woman’s child has a 25% chance of developing the condition. Both of these are extremely high risks compared to the incidence of the disorder in the population so it is inappropriate to suggest that the risk is low.
- A person who has the pathogenic expansion will develop the disorder, and the woman described in this vignette is at 50% risk of having the allele, so it is not appropriate to suggest that she is OK until she develops symptoms.
- The age where myotonic dystrophy manifests in an affected person is dependent on the number of CTG repeats in the gene. Individuals who have a larger number of repeats are likely to have an earlier disease onset and increased symptom severity. The repeat length is unstable and can increase through successive generations, resulting in a decrease in the age at onset, and an increase in the disease severity. This phenomenon is known as anticipation. For this reason, it is not appropriate to estimate onset age or whether she would be affected with mild or severe forms of the disorder.

1. A 56-year-old woman comes to the physician to discuss her risk of developing breast cancer after her 49-year-old sister was diagnosed with breast cancer. Additionally, her mother also had breast cancer that was diagnosed at age 76. The patient had a direct-to-consumer genetic test that shows that she does not have any of the three most common *BRCA1* pathogenic alleles that are known to be present in the Ashkenazi Jewish population. She is relieved, but she doesn’t fully understand the test result and wants help to interpret it. Which of the following statements from the physician would be most useful in helping this woman understand these test results?
2. “A genetic test will not be useful here; we should instead focus on your mammogram.”
3. “It is good news that you don’t have one of these common *BRCA1* pathogenic variants.”
4. “Why did you spend your hard-earned money on a direct to consumer genetic test?”
5. “Regardless of the genetic test, it is likely that you have familial breast cancer.”
6. “There are many possible *BRCA1* variants and a clinical test is better to identify them.”

Answer: E

Rationale:

- There are indeed many *BRCA1* or *BRCA2* variants and a clinically approved genetic test is the best way to ensure that a potential variant will be reported so risk can be optimally interpreted. Even with the best *BRCA1/2* test, there is some uncertainty in the result, and a geneticist, genetic counselor, or an oncologist who is well versed in genetics is best-equipped to discuss the test results with the patient.
- Genetic testing is indeed useful as it might help guide management options and inform familial risk. A mammogram is also important.
- It is relatively meaningless to point out that a person does not have one of the most common *BRCA1/2* pathogenic variants since the actual frequency of these alleles in the population is quite low. Additionally, there are thousands of other possible variants in *BRCA1/2* as well as other cancer-associated genes.
- It is not useful to admonish a person for obtaining a DTC genetic test, she might have obtained this testing for a different reason such as curiosity about ancestry.
- A familial breast cancer susceptibility syndrome cannot be diagnosed simply because a mother and daughter had breast cancer, as most forms of cancer are either sporadic or exhibit familial clustering due to multifactorial inheritance characteristics of the cancer.

1. An 18-year-old man undergoes a physical examination prior to joining an elite training program for swimming. He has been training for more than 10 years to reach this level of competition. An electrocardiogram shows that he has a prolonged QT interval, otherwise he is in good health. During the next office visit, the physician breaks the news of the diagnosis to the athlete who then asks “what do I do now?” Which of the following is the most appropriate response by the physician?
2. “We should surgically implant a cardioverter-defibrillator so you can continue to swim competitively.”
3. “We need to order genetic testing so that we can find out if you have a pathogenic variant”.
4. “The risks associated with your condition will change depending on your lifestyle choices.”
5. “You need to quit swimming because it is one of the riskiest activities you can do”.
6. “You need to tell your family so we can get them all tested to see who else is affected”.

Answer: C

Rationale:

- Here, the best choice is helping the patient consider his options and to understand that his behaviors will affect his risks. Furthermore, he can be reassured that the physician will help to make his choice a safe one.
- One of the safest options for this man is to stop swimming. However, as an elite athlete, he might not want to give up on his dreams of competing at a high level. Pushing the issue, might result in the man continuing training while avoiding surveillance, treatment, and management, resulting in an even greater risk of severe consequences.
- It is not a good option at this point to suggest surgical implantation of a cardioverter-defibrillator (ICD) as the boy has not decided what he would like to do. Furthermore, an ICD is often an option of last resort, and used after other management strategies have been tried without success.
- At this point, it is not appropriate to tell him to obtain genetic testing as the critical issue is minimizing his risks for cardiac complications.
- Likewise, the physician may advise the patient that it is a good idea to tell the other people in his family so they can be tested, but he cannot force him to do so.

1. A newborn boy is born via a spontaneous and uncomplicated vaginal delivery. He has Apgar scores of 7 and 9 with normal vital signs, and his weight is 8 pounds 5 ounces (3.8 kg). Blood is drawn by a heel stick for a newborn metabolic screen at 24 hours of age. Results are returned six days later and show elevated phenylalanine (12.21 mg/dL; normal < 2.00 mg/dL) with depressed tyrosine (0.42 mg/dL; normal < 4.98 mg/dL). The parents are immediately contacted by phone to discuss the test results. Which of the following statements contains the most important point that should be made by the physician at this time?
2. “Let’s wait until we verify the test diagnostically, as many screening tests show false positive.”
3. “Your son has hyperphenylalaninemia so let’s schedule an appointment later this week to discuss the results.”
4. “Elevated levels of phenylalanine imply that your son has PKU, an inborn error of metabolism.”
5. “Since your son might have PKU he must be placed on a strict diet until we verify the result”
6. “This abnormal result means we need to do a follow-up diagnostic test and we’ll go from there.”
7. “Your son has tested positive for a potentially dangerous and uncurable metabolic disorder.”

Answer D

- Although newborn screening for phenylalanine (phe) levels is not considered diagnostic for phenylketonuria (PKU), a positive result is sufficient evidence for this baby to be placed on a phe-restricted diet until the diagnosis is confirmed. A secondary diagnostic test is then ordered to quantitatively measure phenylacetate, phenyllactate and BH_4_ levels to confirm PKU and characterize its causes. In this case, the dietary restriction should begin immediately because there is little harm in beginning the diet. Since elevated phe levels are neurotoxic, there is a high degree of potential harm of not beginning the diet if PKU is confirmed.
- It is true that a positive result on a newborn metabolic screen does not necessarily mean that an infant has a particular metabolic disorder and all positive screen results require follow-up diagnostic testing. The reason a diagnostic test is required is that some infants test positive for elevated phe without having frank PKU.
- A delay in management may result in significant consequences due to the known toxicity of elevated phenylalanine levels. Phe levels should be brought under control immediately until its causes are better understood.
- Suggesting that this is a false positive test because the condition is rare is completely inappropriate.
- Elevated serum phenylalanine might be toxic, and it is an indication of a potential emergency situation – it needs to be acted on immediately. Postponing the discussion of the diagnosis and its implications can result in severe negative consequences for the baby.
- Simply stating that an elevated phe level is an inborn metabolic error, or that the screening result indicates a dangerous situation without providing context or a next step are not the best statements at this time and are preliminary until a diagnostic test is completed.

1. A 28-year-old pregnant woman undergoes prenatal genetic testing at 12 weeks of gestation because she wants to be sure that her baby is healthy. The analysis shows that the fetus has a well-characterized, but rare microdeletion that has been associated with developmental delay and autism. The penetrance of the condition is estimated to be approximately 70%. After informing the woman of the diagnosis, the physician suggests that the parents consider terminating the pregnancy, as they are likely to have a child who will have special needs. Which of the following options best describes the physician’s comments?
2. Directive
3. Sympathetic
4. Empathetic
5. Non-directive
6. Balanced

Answer: A

Rationale:

- Here the physician is providing directive counseling, rather than the more desirable balanced or non-directive counseling. The physician’s own values and beliefs are projected onto the parents, suggesting that they would not want to have a baby who has the potential to have developmental delay or autism. This directive response is particularly problematic in this situation when the penetrance of the condition is known to be approximately 70%, meaning that about 30% of individuals with this microdeletion do not develop the phenotype.
- A more appropriate (balanced or non-directive) response would be to outline all of the options available to the parents, from pregnancy termination to carrying the pregnancy to term and delivering the baby. This would be followed up with a discussion of what developmental delay and autism could look like as the baby grows up.
- The physician is also not expressing either sympathy or empathy. A sympathetic response would reinforce the negative connotations of the condition and is inappropriate.
- An empathetic response would be appropriate, recognizing the difficult situation that the parents are currently facing, and providing support for them as they come to a decision that works for them.
